# 3D cancer models: One step closer to *in vitro* human studies

**DOI:** 10.3389/fimmu.2023.1175503

**Published:** 2023-04-11

**Authors:** Nicoletta Manduca, Ester Maccafeo, Ruggero De Maria, Antonella Sistigu, Martina Musella

**Affiliations:** ^1^ Dipartimento di Medicina e Chirurgia Traslazionale, Università Cattolica del Sacro Cuore, Rome, Italy; ^2^ Fondazione Policlinico Universitario ‘A. Gemelli’ - Istituti di Ricovero e Cura a Carattere Scientifico (IRCCS), Rome, Italy

**Keywords:** tumor microenvironment, cancer model, spheroids, organoids, microfluidic devices, organ-on-a-chip, drug screening

## Abstract

Cancer immunotherapy is the great breakthrough in cancer treatment as it displayed prolonged progression-free survival over conventional therapies, yet, to date, in only a minority of patients. In order to broad cancer immunotherapy clinical applicability some roadblocks need to be overcome, first among all the lack of preclinical models that faithfully depict the local tumor microenvironment (TME), which is known to dramatically affect disease onset, progression and response to therapy. In this review, we provide the reader with a detailed overview of current 3D models developed to mimick the complexity and the dynamics of the TME, with a focus on understanding why the TME is a major target in anticancer therapy. We highlight the advantages and translational potentials of tumor spheroids, organoids and immune Tumor-on-a-Chip models in disease modeling and therapeutic response, while outlining pending challenges and limitations. Thinking forward, we focus on the possibility to integrate the know-hows of micro-engineers, cancer immunologists, pharmaceutical researchers and bioinformaticians to meet the needs of cancer researchers and clinicians interested in using these platforms with high fidelity for patient-tailored disease modeling and drug discovery.

## 1 Introduction

Despite the impressive progress in early detection and development of increasingly efficient and tumor-targeted treatments over the past decade, cancer remains a major burden of disease worldwide and one of the leading causes of death (1). Currently, the greatest challenge in oncology is to move away from old “one-size-fits-all” treatments, which, in the majority of cases, work well only for a few patients, toward novel personalized “one dose-one patient” therapeutic approaches (2).

Tumor heterogeneity, within and across cancers, often represents the most significant roadblock in the implementation of effective patient-specific therapies (3–5). Of note, clinical diagnoses are mainly based on tumor biopsies which do not really capture the extensive intratumoral heterogeneity but may hide newly emerging, highly aggressive, tumor clones. Moreover, patients with the same cancer subtypes often present different tumor phenotypes that dynamically evolve during disease progression and clinical treatment, and lead to the most disparate therapeutic responses, including natural and acquired therapeutic resistance (6, 7). It is now well established that tumors are not simple masses of neoplastic cells, but rather heterogeneous collections of infiltrating or resident host non-neoplastic cells [mainly T and B lymphocytes, natural killer (NK) cells, dendritic cells (DCs), monocytes, endothelial cells, perycites, cancer-associated fibroblasts (CAFs), mesenchymal stromal cells and adipocytes], niche-relevant soluble factors (*i.e.*, cytokines, growth factors, metabolites, enzymes, miRNAs) and altered extracellular matrix (ECM) that actively interact with one other and constitute the tumor microenvironment (TME) (8). Increasing evidence highlights that this evolving and reciprocal interplay between cancer cells and TME players is a disease-defining factor as it governs cancer initiation, metastasis and drug resistance and thus represents a promising therapeutic target (9, 10).

In light of this, the chance to achieve the designing of successful personalized anticancer strategies, characterized by more durable and side effect-limited (or even better free) responses, will depend on the ability to accurately model cancer heterogeneity and TME interactions (11). If on the one hand two dimensional (2D) cultures, xenografts and syngeneic mouse models have made the history in cancer research, on the other hand, to date scientists are addressing their focus more and more on three dimensional (3D) *in vitro* systems which can preserve tumor proper genetic, proteomic, morphological and pharmacotypic features while offering the unprecedented possibility to deeply dissect tumor-stroma dynamics.

**Figure 1:**
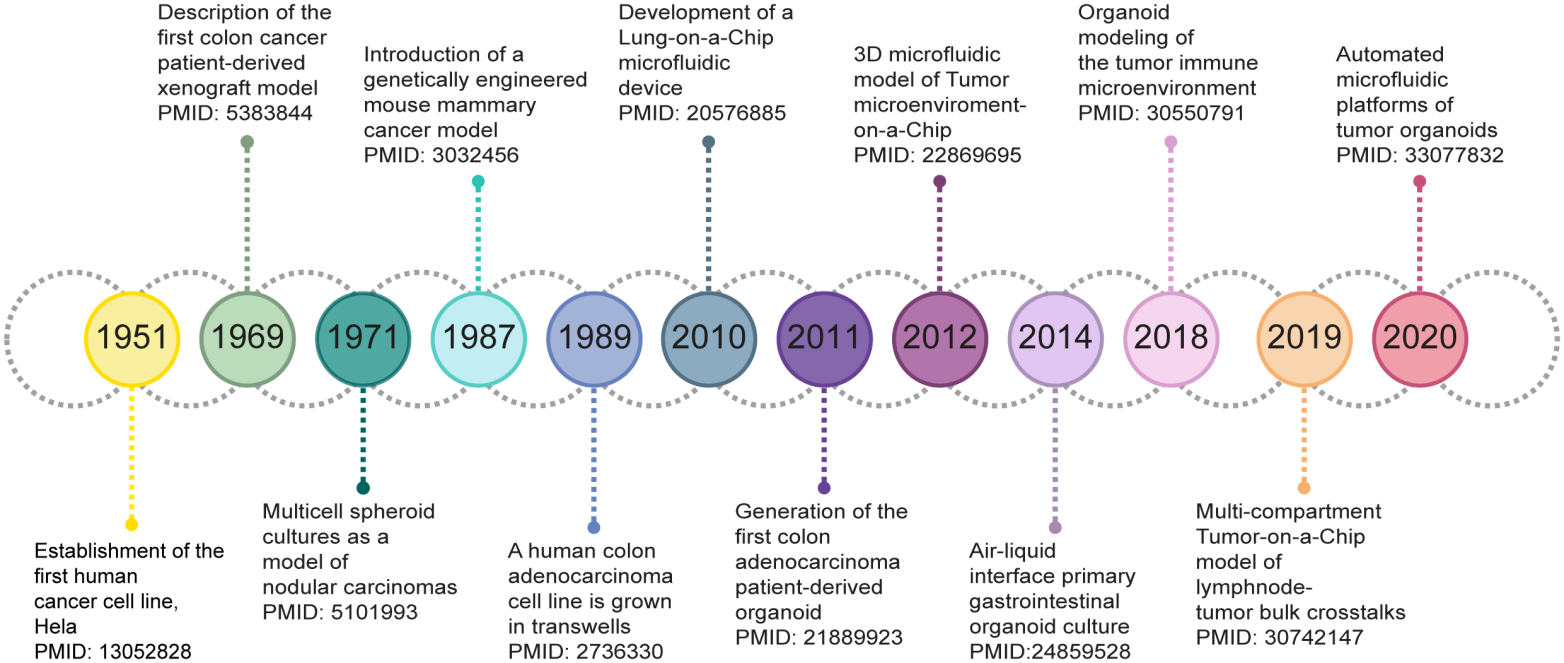
Timeline of milestones in the development of 3D cancer models.

In this review we present an overview of cancer model (r)evolution over the years (**Figure 1**) for studying the biological implications of the TME on cancer progression and response to therapy. We critically discuss the opportunities of state-of-the-art *in vitro* 3D cell culture strategies, with an emphasis on cancer spheroids, organoids and Tumor-on-a-Chip (ToC) models, for the development of microphysiological platforms recreating human cancers growing within living organs. In addition, we point out the current limitations and challenges that such novel culture systems should overcome to fully establish, validate and exploit the fidelity of 3D models for cancer research and clinic.

## 2 Chronicles of conventional cancer models in preclinical research

For many years, *in vitro* 2D cell cultures and *in vivo* xenografts or genetically engineered animal models have been the gold standards in cancer research. Nevertheless, these “conventional” models lack the ability to sustain the complex genetic and phenotypic heterogeneity of the respective human patient-derived tumor samples as well as to model the disease pathogenesis while simultaneously facilitating comprehensive cellular and environmental manipulation (11). Given their wide availability, reproducibility, high-throughput and the overall low cost, 2D monolayer cultures of immortalized cell lines have been widely employed as initial screening models to elucidate the mechanisms of cancer biology and to identify the efficacy and safety of several drug candidates (12, 13). However, a large body of evidence indicates that these systems still present several drawbacks. First, isolation and culture maintenance of cancer cell lines from patient biopsies may be tricky and unproductive. Second, once cultured, these cells adhere, spread, and grow on a flat synthetic surface, do not conserve the original morphology and polarization, and therefore can potentially lose crucial cellular signaling pathways or change their responses to external *stimuli* (14–16). Third, cells in 2D cultures commonly undergo to extensive clonal selection thus resulting in the establishment of derived cell lines which no longer recapitulate the genetic heterogeneity of parental tumors. Finally, *in vitro* cancer cell lines are rarely flanked by a patient-matched 2D normal tissue counterpart and, most importantly, they do not provide significant information about the intricate network of dynamic interactions within the 3D TME of living patient’s tumors, which instead can dramatically affect the efficacy of cancer therapies (17–19). In an attempt to partially simulate the complex *in vivo* cell-cell communications occurring in the TME, 2D co-cultures of cancer cell lines and different types of exogenous and heterogeneous cells [such as peripheral blood mononuclear cells (PBMCs) or CAFs] have been set up (20). In this regard, transwell cell cultures have been exploited to assess the capability of cells to migrate toward a particular chemo-attractant and additionally to test the ability of cancer cells to invade and bypass the ECM and to extravasate by pre-coating the top of the membrane insert with thin layers of ECM gels (such as collagen or Matrigel™) and endothelial cells (21). Anyway, despite somewhat more complex, such 2D reconstituted systems failed to faithfully model primitive intrinsic tumor stroma and its 3D architecture (22, 23).

Otherwise, preclinical *in vivo* animal models, such as patient-derived xenografts (PDXs) and genetically engineered mouse models (GEMMs), enable unique studies that intrinsically contemplate 3D tumor tissue organization and therefore offer system-level analysis of tumor onset, progression and treatment response (24, 25).

Due to their ability to retain morphologies, architectures and molecular signatures very close to those of the original tumors, PDX mouse models provide promising platforms for personalized cancer medicine (26). Hence, they have been increasingly utilized in both basic and preclinical cancer research as potential tools for biomarker detection, drug screening, drug-resistance mechanism investigation and novel therapy development (27–30). PDXs are generated by transplanting subcutaneously or orthotopically freshly derived patient material into immunodeficient mice. Even though subcutaneous transplantation models allow for easier cell transfer and precise monitoring of tumor formation and growth, orthotopic PDX mouse models better mimic the biological characteristics of the donor tumor in terms of phenotype - cancer heterogeneity and behavior - metastatic potential (31–34). Nevertheless, some important and unavoidable limitations have restricted PDX application in precision cancer therapy. Since they rely on immunocompromised/immunodeficient mice that lack the adaptive immune system, PDX mouse models do not fully recapitulate the surrounding tumor stroma and thus constitute inappropriate tools for the screening and the functional analysis of new immunotherapeutic agents (35). Furthermore, the progressive replacement of human stromal cells with recipient mouse cells may affect drug response predictions (36). In the last years, new humanized PDX mouse models have been generated by engrafting patient-derived tumors into immunodeficient mice bearing CD34+ human hematopoietic stem cells or PBMCs, but cost, time, throughput, and complete immune compatibility, remain unmet challenges (37, 38). Undoubtedly, the main weakness of PDX models is the inability to graft all tumor subtypes. For instance, hormone-sensitive breast cancer has a lower rate of engraftment than triple-negative tumors (39) and, more generally, non-metastatic tumors fail to stably engraft and grow in mice (32, 40). Finally, PDX models suffer from clonal selection pressure upon human tumor tissue engraftment and propagation leading to genetic and phenotypic divergence from the parental tumor (41, 42).

By contrast, GEMMs develop *de novo* tumors in an immunoproficient microenvironment thus enabling the investigation of the native interactions between cancer cells and the surrounding TME and representing valuable tools for testing the potential of cancer immunotherapies (43). Additionally, tumors arising in next-generation GEMMs closely mimic the histopathological and molecular features of their human counterparts, display genetic heterogeneity, and are able to spontaneously progress toward metastatic disease (44, 45). Although GEMMs have been successfully used in preclinical research (as reviewed in (46)**)** to validate candidate cancer genes and drug targets, assess therapy efficacy, dissect the impact of the TME, and evaluate mechanisms of drug resistance, there are still some aspects that need to be improved. In particular, their overall genetic manipulation is relatively limited and the introduction of novel (non)-germline mutations is a laborious and slow process (24).

On the whole, the development and validation of PDX and GEMM models is expensive, time- and resource-consuming, relatively low-throughput and subject to increasing ethical pressure for replacement solutions according to the 3Rs’ principle in animal experimentation (47). As a result of these limitations, even preclinical *in vivo* models generally have a dramatic poor performance (~3%) in terms of predicting the clinical success of next-generation anticancer therapies (48).

## 3 3D models: Bridging the gap between cell cultures and live tissues

**Figure 2:**
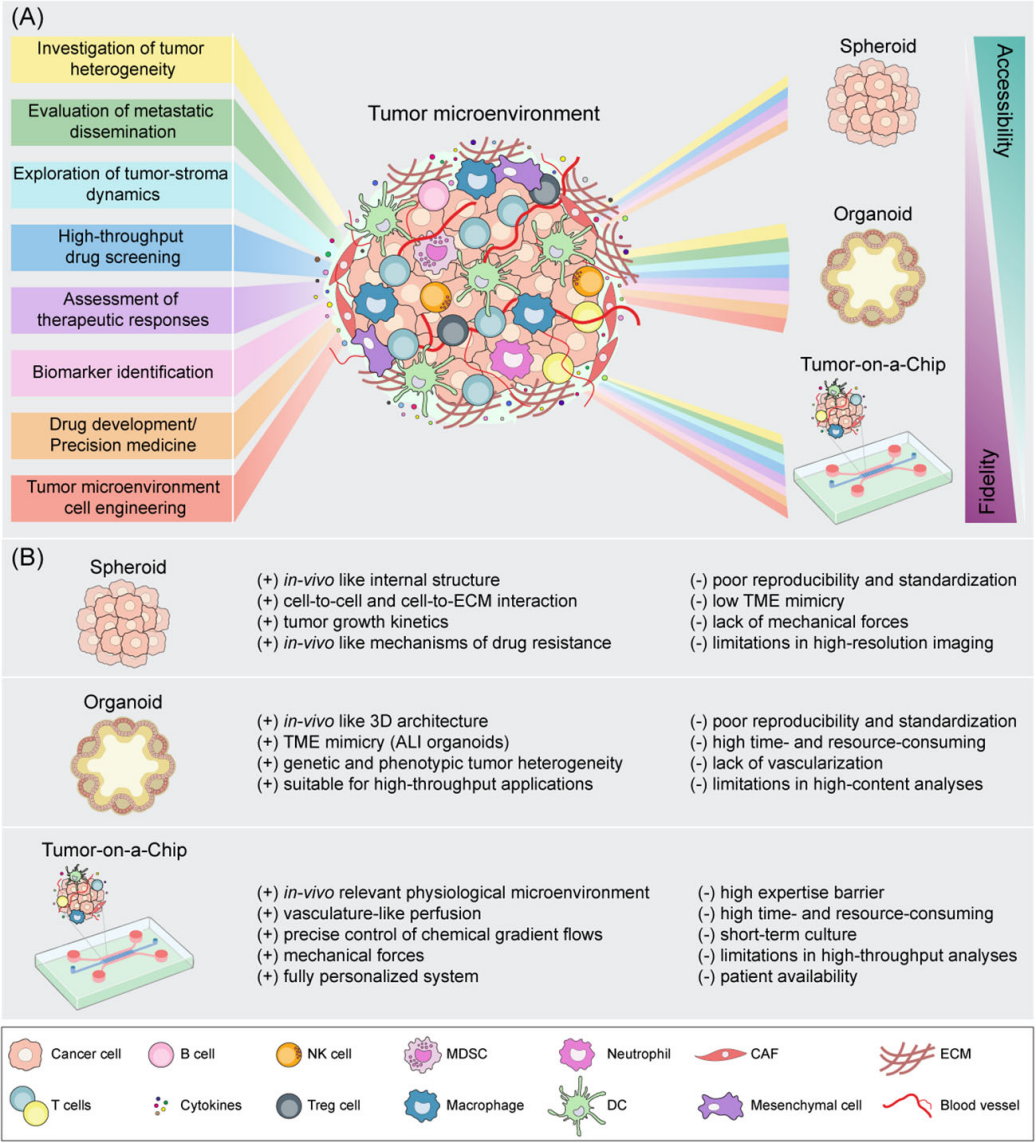
Schematic representation of spheroid, organoid and Tumor-on-a-Chip cell culture strategies for tumor microenvironment mimicking. **(A)** Downstream applications of state-of-the-art 3D models according to their accessibility and biological fidelity. **(B)** Benefits (+) and drawbacks (-) of 3D *in vitro* models. CAF, cancer-associated fibroblast; DC, dendritic cell; ECM, extracellular matrix; NK, natural killer; MDSC, myeloid-derived suppressor cell; Treg, regulatory T cell.

The need to reduce drug failure in clinical trials has encouraged researchers to deploy more sophisticated *in vitro* surrogate systems which can recreate human organs and diseases in the laboratory bench. In recent years, 3D cell models have gained even more attention in cancer research for their ability to closely replicate several hallmarks of *in vivo* tumors. Indeed, unlike 2D cell cultures, such systems provide a more realistic insight of tumor-tissue architecture, multicellular complexity and dynamic interplay between cancer cells and TME thus holding the great promise for many applications in tissue engineering, drug development, and precision medicine (49, 50). In the following sections, we will explore, in order of biological and technological complexity, the characteristics and potential applications of the most cutting edge 3D systems (**Figure 2**).

### 3.1 Tumor spheroids

Spheroids are one of the best established 3D culture methods for the study of tumor biology (51, 52). As extensively reviewed in (53), spheroids are microsized aggregates of closely-packed cells which accurately recapitulate some important features of solid tumors including internal structure, cellular heterogeneity, cell signaling pathways, ECM deposition, cell-to-cell and cell-to-ECM interactions, growth kinetics, gene expression and drug resistance. These unique characteristics highlight the potential of spheroids to be used as suitable *in vitro* tools for high-throughput screening of anticancer therapeutics (54–56).

Depending on cellular source and preparation protocols, spheroids can be distinguished into four major types, namely: (*i*) multicellular tumor spheroids (MCTSs) assembled using primary cell or cell line suspensions, (*ii*) tumorospheres obtained from solid tumor dissociation, (*iii*) tissue-derived tumorospheres generated from tissue remodeling after partial enzymatic or mechanical dissociation and (*iv*) organotypic multicellular spheroids consisting of cut and minced tumor fragment cultures obtained without dissociation (57). Of these, MCTSs are the best characterized spheroid models and have been widely used to reproduce different solid tumors, such as breast (58), cervical (59), colon (60), lung (61), pancreas (62), and prostate cancer (63), among the others. Currently, multiple techniques, requiring or not the incorporation of an exogenous scaffold, are available for MCTS production (64). In the most commonly employed scaffold-free methods, cells are cultured in conditions that force to strong cell-to-cell interactions and ultimately support cancer cell aggregation and ECM deposition. Several anchorage-independent methodologies have been developed, including the noteworthy hanging drop and liquid overlay protocols, followed by agitation-based, microencapsulation, and magnetic levitation systems (reviewed in detail in (65–67)). By contrast, scaffold-based approaches enable cells to grow dispersed on hydrogels that mimic ECM architecture or anchored to acellular matrices, which may be comprised by natural (*e.g.*, alginate, chitosan, dextran, hyaluronic acid), synthetic *(e.g.*, poly lactic-co-glycolic acid, polycaprolactone, polyvinyl alcohol and polyethylene glycol) biomaterials or decellularized natural ECM (*e.g.*, Matrigel™, collagen, fibrin, gelatin) (50, 68–70). More recently, advances in bioengineering techniques have emphasized the role of microfluidics and 3D bio-printing for the development of more complex tumor spheroids with well-defined architecture, composition and high reproducibility which can model various cancer types and stages (71–74). Intriguingly, such Spheroid-on-a-Chip technologies have been proposed as preclinical platforms to investigate tumor angiogenesis, metastatic potential and chemotherapy response in glioblastoma, breast adenocarcinoma and ovarian cancer (73, 75, 76), as detailed below.

MCTCs have the ability to mimic to a great extent the TME thus offering a good representation of oxygen, nutrient, and other soluble factor diffusion and exchange (77). Indeed, if cells grown in monolayer cultures are uniformly exposed to nutrients and oxygen, cancer cells cultured as spheroids instead experience physiochemical gradients similar to those observed in micrometastases and avascular tumors (77). Moving toward the center of the spheroid, oxygen nutrient and pH levels decrease, whereas the amounts of carbon dioxide, lactate and waste products increase. Owing to the limited diffusion of nutrients and oxygen, larger spheroids (>500 µM in diameter) display an internal structure consisting of different cell layers: an inner anoxic and acidic core containing necrotic cells, a middle hypoxic zone of quiescent/senescent cells and an outer layer of highly proliferating cells (55, 78–80). Such heterogeneous multilayered organization is reported to be the key factor behind the use of spheroids as preclinical models to evaluate the therapeutic efficacy of anticancer treatments, including chemotherapy, radiation therapy, and immunotherapy (81–84).

The hypoxic environment found in the core of the spheroids is detrimental for all those drugs known to induce DNA and membrane damage *via* production of reactive oxygen species (*e.g.*, 5-fluorouracil, cisplatin, doxorubicin, and irinotecan) (77, 85, 86). Accordingly, Doublier and co-workers observed that in estrogen receptor-positive MCF7 breast cancer spheroids activation of hypoxia-inducible factor 1 together with an increase of P-glycoprotein expression were responsible for doxorubicin resistance (87). Similarly, Kim and colleagues showed that U251 glioma and U87 astrocytoma cells, grown as spheroids under hypoxia conditions, exhibited increased apoptosis resistance upon exposure to doxorubicin and the caspase-3 activating molecule resveratrol, as compared to monolayer cell cultures (88). Additionally, senescent and necrotic cells that reside in MCTS’ inner zones were shown to be more resistant to antiproliferative compounds (*e.g.*, carboplatin, cisplatin, doxorubicin, oxaliplatin, methotrexate, and paclitaxel) than rapidly dividing cells (89, 90). In this regard, different breast cancer cell lines (*i.e.*, BT-549, BT-474, and T-47D) exhibited greater resistance to doxorubicin and paclitaxel associated with higher levels of hypoxia, increased percentages of G0-dormant cell subpopulation and lower expression of cleaved-PARP and caspase-3, when cultivated as 3D MCTSs. Moreover, the peculiar acidic pH of the spheroid core can induce changes in the net charge of some chemotherapeutics (*e.g.*, melphalan, methotrexate, mitoxantrone and vinca alkaloids) thus negatively affecting their intracellular uptake (91–93).

Importantly, the deposition of ECM proteins and the close ECM-cells and cell-cell physical interactions are known to increase spheroid density, leading to a higher interstitial fluid pressure which is responsible for the impaired penetration of anticancer drugs (94, 95). Therefore, compact and larger MCTCs are often more resistant to both chemotherapy and radiotherapy than loosely aggregated cells (96, 97). Of note, due to their structural similarities with *in vivo* solid tumors, MCTCs may also be used to improve the predictive value of nanomedicine screening and their physicochemical properties, by modeling the selective penetration, accumulation, retention, and distribution of drug-loaded nanocarriers within the tumor mass (98, 99).

MCTCs can be constituted exclusively of cancer cells (homotypic spheroids) or of cancer cells co-cultured with other cell types (heterotypic spheroids) such as fibroblasts, endothelial cells or immune cells (53, 58). Such heterotypic MCTCs are shown to be extremely helpful for studying tumor-immune system interactions and testing immunotherapeutic agents. Intriguingly, Coureau et al. recently exploited *in vitro* heterotypic co-cultures of human colorectal cancer MCTSs with immune cells to assess the infiltration, activation and function of T and NK cells. They showed that allogeneic T and NK cells infiltrated cell line-derived spheroids, inducing immune-mediated cancer cell killing and 3D structure destruction *via* the engagement of the activating receptor NKG2D (100) while cancer cells tried to evade immune recognition by upregulating HLA-E, ligand of the inhibitory receptor NKG2A expressed by CD8 T and NK cells. The simultaneous antibody targeting of both NKG2D ligands on cancer cells, in order to elicit an antitumor antibody-dependent cellular cytotoxicity (ADCC), and of the inhibitory receptor NKG2A on immune cells, highlighted an increased immune cell infiltration as well as a greater antitumor response (100). Ultimately, the authors confirmed these results in autologous co-cultures of colorectal cancer patient-derived spheroids and tumor-infiltrating lymphocytes (TILs) to generate a clinically relevant functional assay to the study of immunotherapies (100). A heterotypic colon carcinoma spheroid model was also used to evaluate the anticancer immune response of allogeneic Vδ2 γδ T lymphocytes triggered by zoledronate or cetuximab (101). Furthermore, gastric and ovarian MTCS models have been exploited to test the therapeutic efficacy of chimeric antigen receptor (CAR) T cells targeting the mesothelin antigen (102), whose preclinical and clinical testing in combination with immune checkpoint blockade (ICB)-based therapy has been extensively discussed in (103–105). Of interest, Dordick’s group have recently developed a high throughput 3D tumor spheroid microarray consisting of a 330 micropillar-microwell sandwich platform where NK cells are co-cultured with pancreatic (MiaPaCa-2) or breast cancer cell lines (MCF-7 and MDA-MB-231) to faithfully recapitulate the hypoxic TME and investigate NK-cell mediated cell cytotoxicity in combination with the two monoclonal antibodies Trastuzumab and Atezolizumab (106).

Overall, these models are limited by the absence of stromal cells, which are usually present in the TME and are critical to the establishment of a chemoresistant cancer cell niche (107). Driven by the enticing possibility of improving cellular heterogeneity in MCTS cultures, Jeong et al. established a more clinically relevant colorectal cancer model by combining 3D co-culture with microfluidic technology. Specifically, tumor spheroids were grown within a collagen matrix-incorporated microfluidic chip and co-cultivated with CAFs in a microscale distance away, allowing mutual microenvironmental interactions culminating in CAF activation, as demonstrated by the increase of α-smooth muscle actin (α-SMA) expression and migratory activity, as well as the induction of resistance to the chemotherapeutic paclitaxel (108).

To further complicate the system, a scaffold-free MCTS consisting of a triple co-culture of pancreatic cancer cells (PANC-1), fibroblasts (MRC-5) and endothelial cells (HUVEC) was assembled to closely mimic the *in vivo* influence of the surrounding TME on cancer cell therapeutic resistance (109). Remarkably, a heterotypic spheroid model composed of tumor cells, fibroblasts, and immune cells was developed to assess the efficacy of novel cancer immunotherapy agents [*i.e.*, IL-2 variant and tumor- or fibroblast-targeted T cell bispecific antibody] both as monotherapy and in combination (110). To better understand the role of macrophages in the TME using spheroids, Rebelo et al., developed a 3D culture model based on alginate microencapsulation and stirred culture strategies which enclosed tumor cell spheroids of non-small cell lung carcinoma (NSCLC), CAFs and monocytes. In such a way, they successfully recreated an immunosuppressive TME enriched in cytokines/chemokines (IL-4, IL-10, IL-13, CCL24, CXCL1), ECM elements (collagen type I, IV and fibronectin) and metalloproteases (MMP1/9), supporting cell migration and monocyte polarization toward an M2-like macrophage phenotype (109, 111). Similarly, Kuen et al., established pancreatic cancer spheroids consisting of different cancer cell lines (PaTu-8902, BxPc3, HPAC, and MiaCaPa-2) and MRC-5 fibroblasts, which then incubated with peripheral blood-derived monocytes. Such monocytes were able to penetrate into the spheroids, reflecting the *in vivo* tumor infiltration, and differentiated into M2-like macrophages (112).

Despite their huge potential, several issues still exist which hinder the application of MCTSs as high-fidelity preclinical cancer models. The main challenges concern the lack of standard protocols and methods to establish spheroids of uniform size and shape. In addition, some techniques are associated with low-throughput and difficulty in retrieving cells for readout analysis. Indeed, if on the one hand homotypic MCTS models provide a too simplistic tissue representation, on the other hand they are more suitable for high-throughput screenings. Conversely, heterotypic MCTSs strengthen the *in vitro* representation of TME but requires a mindful optimization of the cellular composition in terms of cell ratios and cell media components, consequently affecting the throughput (113). Furthermore, spheroids do not reproduce the complexity observed in the 3D tissue architecture of living organs nor incorporate mechanical forces (such as fluid shear stress, hydrostatic pressure and tissue deformation) that can significantly influence cancer cell behavior (114). Although nowadays a plethora of techniques are commonly employed to perform phenotypic and genetic analysis of tumor spheroids, such experimental procedures conceal several drawbacks. For instance, standard biochemical assays to evaluate viability and cytotoxicity (such as the acid phosphatase activity, the MTT, the Trypan Blue exclusion, and the lactate dehydrogenase assay) were found to be inefficient in 3D spheroids, usually due to the incomplete probe penetration and limited sensitivity (115–118). Optical, phase contrast, confocal, fluorescence and electron microscopic techniques are reported to be particularly valuable for characterizing spheroid size, morphology and internal organization (119–121). However, 3D model imaging is generally affected by poor light penetration, light scattering by cells, and high background (117, 118, 122). Flow cytometry and western blotting application on 3D structures can also be challenging. Indeed, both the techniques require spheroid enzymatic dissociation into single cell suspensions which inevitably leads to the loss of important information on marker spatial distribution (123–125).

To date, considerable efforts are being made to improve large-scale production of spheroids under highly reproducible conditions and to further adapt quantitative analysis and imaging techniques to such 3D models, in order to extract significant biological data and allow for high-throughput screening of anticancer drugs.

### 3.2 Tumor organoids

Organoids originally arise as 3D *in vitro* stem cell derived cultures that recapitulate the cellular variety, architectural organization and function of their *in vivo* normal tissue counterparts and have the ability to self-organize and self-renew (126–128). Since their discovery, organoids represented an ideal model for studying organ development (129) and host-pathogen interactions (130) by bridging the gap between *in vivo* animal models and *in vitro* 2D cell culture systems. The first attempts of generating organ-specific models *in vitro* date back to the early 2000’s, when Sasai and colleagues demonstrated that embryonic stem cells could differentiate and self-assemble into 3D apico-basally polarized cerebral cortical tissues (131). Shortly after, Sato et al. established gut organoids from single mouse adult intestinal stem cells in specific culture conditions mimicking the *in vivo* stem cell niche and favoring the dynamic proliferation and differentiation of the intestinal crypt epithelium (132). This seminal work paved the way to grow other organotypic cultures of multiple mouse and human epithelial tissues, including colon (133), pancreas (134), liver (135), prostate (136–138), stomach (139), lung (140), endometrium (141), fallopian tubes (142), taste buds (143), salivary and mammary glands (144, 145), retina (131) and brain (131, 146).

Over the years, organoid technology promptly adapted to tumor biology providing a novel low-cost approach for cancer modeling and therapy development. Since they usually derive from one or few cells, and follow the different stages of cancer development, tumor organoids preserve key histopathological, genetic and phenotypic features of the parent tumor and retain cancer cell heterogeneity to a greater extent (126, 127, 147). Therefore organoids emerge as promising research tools to improve translational research and may have a potential relevance in clinical decision making (148).

To date, cancer organoids may be generated by multiple strategies. On the one hand genetic engineering of organoids from wild-type tissues or induced pluripotent stem cells provides a unique opportunity for determining the mechanisms of cancer initiation and progression in specific organs, the tumor niche factor requirements and the mutation pattern-related cellular response to anticancer therapies (149–151). Starting from available healthy human tissue-derived organoids, different reports exploited CRISPR-Cas9 genome editing to introduce combinations of common driver mutations and model the multi-hit oncogenic transformation in colorectal (151–153), brain (154), gastric (155), pancreatic (156) and breast (157) cancer. Of note, numerous studies have focused on genetically engineered colorectal cancer organoids carrying oncogenic mutations in Wnt, EGFR, TP53 and TGFβ/BMP signaling pathways to gain deeper insights into the metastatic dissemination program. Surprisingly, although such organoids efficiently grew *in vivo* as invasive tumors, only when transplanted into their orthotopic environment, they were able to develop primary tumors that spontaneously formed distant liver and lung metastases as result of progressive loss of stem cell-niche dependency (158, 159). Organoids were also used to investigate the contribution of cancer stem cells (CSCs) to colorectal cancer clinical progression. Intriguingly, two seminal works from de Sauvage and from Sato teams, demonstrated how the selective CSC depletion restricted primary tumor growth but did not result in tumor regression, owing to the extensive cellular plasticity of human colorectal cancer cells. Indeed, proliferative differentiated cancer cells constantly attempt to replenish the CSC state leading to rapid tumor recurrence upon treatment cessation (160, 161). Remarkably, tumor organoids have also been derived from transgenic mouse strains to study the effects of a particular oncogenic mutation in the context of a specific genetic background. In this regard, Kuo and colleagues demonstrated that TGFβ receptor 2 was implicated in metastatic gastric cancer (162), whereas Fearon’s group showed that the transcription factor CDX2 and BRAFV600E mutations cooperated to promote serrated colorectal cancer development (163).

Given the ability of genome-editing technologies to repair disease-causing genes, as previously demonstrated for the mutated dysfunctional CFTR allele in intestinal stem cell organoids of cystic fibrosis patients (164), genetic engineered cancer organoids are now revolutionarily investigated to test the possibility of reverting particular oncogenic mutations and the so leaded tumorigenic phenotype. Although cancer is genetically much more complex, with tumors typically harboring hundreds of mutations, it was shown that restoration of APC expression recovers crypt homeostasis in a colorectal cancer mouse model and derived organoids (165).

On the other hand, a large body of evidence has provided a proof-of-concept for generating patient-derived organoids (PDOs) which have shown relevant phenotypic and genetic resemblances with their original tumor specimens (166–168) and a tremendous potential in personalized cancer therapy (169). Unlike conventional cancer models, PDOs can be robustly propagated from a small sample size derived from solid/liquid biopsies or surgical resections of primary tumors (167), circulating cancer cells (170) and metastatic lesions (168, 171).

Since the establishment of the first colon adenocarcinoma PDO by Sato et al. (133), long-term tumor organoid cultures were successfully generated from a wide range of other primary colon (167, 172), oesophagus (173), pancreas (174), lung (175), stomach (176), liver (177), ovarian (178), breast (145), brain (179) and prostate (170, 180) cancer tissues, as well as from urothelial (181) and renal carcinoma (182). Importantly, the success rate of organoid generation from these selected cancer subtypes was almost always reported to be >70% and notably higher than that for traditional cancer cell lines (~20–30%) (183). Moreover, follow-up analyses of such 3D models suggest that organoids have the ability to preserve long-term parent tumor’s biology including (epi)genetic, proteomic, morphological and pharmacotypic features. In addition, as PDOs are relatively easy to establish and cheap to maintain, they are suitable for high-throughput applications in the context of precision cancer treatments and help predict treatment responses and stratify individual patients to specific therapeutic regimens.

Therefore several “living biobanks” of PDOs capturing the histological and mutational heterogeneity of human cancers (like colon, pancreas, breast, prostate, liver, lung, stomach, ovary, kidney, bladder, and brain, among the others) have been created in recent years providing a representative collection of well-characterized models for preclinical drug screening and for predicting patient outcomes, as extensively discussed by others (147, 184). In 2015 van de Wetering et al. created the first organoid biobank from colorectal cancer patients consisting of 20 primary tumors matched with adjacent normal-tissue derived organoid cultures. By developing a robotized high-throughput drug screening, they tested 83 compounds (including standard-of-care chemotherapeutics and new targeted inhibitors) across the organoid panel and correlated drug sensitivity with cancer genomic features to identify molecular signatures and clinically relevant biomarkers associated with drug responses (167). In line with this previous report, Sato and colleagues generated a larger biobank of 55 colorectal cancer organoids derived from different histological subtypes and clinical stages, including the poorly differentiated adenocarcinoma, mucinous adenocarcinoma, and neuroendocrine carcinoma and observed a progressive decrease in niche factor requirements during adenoma-carcinoma transition, reflecting accumulation of multiple mutations (172). Interestingly, the authors underscored cancer organoids’ ability to model distinct histopathological features and genetic signatures of their parental tumor counterparts also following xenotransplantation under the kidney capsule of immunodeficient mice, suggesting that such 3D culture systems can be effectively employed to validate *in vitro* drug responses in a more complex *in vivo* environment (172). Similarly, Ooft et al. derived a collection of PDOs from metastatic colorectal cancer patients to predict responsiveness to standard-of-care chemotherapy (185). These organoids were able to predict responses of the biopsied lesion in more than 80% of patients treated with irinotecan-based therapies without misclassifying patients who would have benefited from treatment. Conversely, such predictive value was not identified for 5-fluorouracil or oxaliplatin combined treatment, probably because of the lack of the surrounding TME, which might influence the efficacy of one treatment more than the other. Additionally, to test the potential of organoids to evaluate drug responses in preclinical settings, Verissimo et al. utilized a colorectal cancer organoid panel to evaluate the effect of different RAS pathway inhibitors that are currently used in the clinic, either as single agents or in combinations. Using this strategy, the authors confirmed that the presence of mutant RAS strongly correlated with resistance to these targeted therapies. Moreover, they highlighted that combinatorial targeting of the EGFR-MEK-ERK pathway in RAS mutant organoids effectively suppressed tumor growth by inducing a transient cell-cycle arrest rather than cell death (186). Moreover, Ganesh et al., established a biorepository of 65 patient-derived rectal cancer organoid cultures from patients with primary, metastatic or recurrent disease to study individual responses following chemoradiation (187).

As pancreatic cancer is one of the most lethal malignancies with high recurrence rate and a minor survival benefit following systemic therapy, different libraries of primary pancreatic ductal adenocarcinoma PDOs were generated to determine prognosis-predictive gene expression signatures (156, 174). Notably, Tiriac et al., attempted to fully recapitulate the mutational spectrum and transcriptional subtypes of primary pancreatic cancer and hence established pancreatic cancer organoids from a comprehensive cohort of 138 patients. Detailed pharmacotyping of these organoid lines revealed genetic and transcriptomic signatures associated with anticancer drug response that could potentially correlate with patient clinical outcomes. Interestingly, by focusing their attention on 9 patients with advanced pancreatic adenocarcinoma, they obtained a retrospective clinical follow-up which perfectly matched with PDO chemosensitivity profile (174). Recently, a living biobank of more than 100 breast cancer organoids was generated from a wide variety of primary and metastatic tumors broadly recapitulating the diversity of the disease. Besides preserving the typical breast cancer morphology and histopathology, most of these organoids also retained the hormone receptor and the HER2 status of the original tumors allowing *in vitro* drug screens that were consistent with patient response (145).

Alongside these large biobanks, smaller PDO collections from advanced prostate and primary liver cancer were generated that helped validate that tumor organoids recapitulate molecular and genomic diversity of cancer subtypes and enable physiologically relevant drug screens (170, 177).

Kim et al. reported a method for successfully creating a living biobank of 80 lung cancer organoids that were assessed for drug sensitivity to both cytotoxic (*i.e.*, docetaxel) and targeted agents (*i.e.*, olaparib, erlotinib and crizotonib). According to what observed in patients, organoids exhibited a mutation-based drug sensitivity profile. Therefore, as expected, responses to olaparib (PARP inhibitor), erlotinib (EGFR tyrosine kinase inhibitor) and crizotonib (c-Met inhibitor) correlated with BRCA2, EGFR and MET mutational status, respectively (175). Moreover, Vlachogiannis et al., applied PDOs to predict the clinical outcomes of gastrointestinal cancer patients undergoing a compound library of drugs (encompassing chemotherapeutics, immunotherapeutics and targeted therapy agents) either already approved in the clinic or currently in clinical trials. By comparative analysis of the drug sensitivity of patients with metastatic gastrointestinal cancers and that of corresponding PDO models, they showed that the PDO model can accurately recapitulate patient responses in the clinic and could be implemented in personalized medicine programs to define cancer vulnerabilities while improving treatment responses (171). Two organoid platforms that capture intra- and interpatient heterogeneity were also successfully developed from multiple stages and subtypes of ovarian cancer. PDO drug screening of both chemotherapeutics (platinum/taxanes) and targeted agents (PIK3K/AKT/mTOR inhibitors or PARP inhibitors) revealed relevant differences in drug sensitivity which significantly correlated with clinical responses (188, 189).

In 2020, Calandrini et al. described the first pediatric cancer organoid biobank consisting of tumor and matching normal organoid cultures from over 50 children with different subtypes of kidney cancer, including Wilms tumors, malignant rhabdoid tumors, renal cell carcinomas, and congenital mesoblastic nephromas. By using this approach, they identified treatments with the best therapeutic ratio, considering both tumor efficacy and normal tissue toxicity (190). Yao et al. established a living organoid biobank of locally advanced rectal cancer and showed that PDOs could predict chemoradiation responses in patients (191). Yet, Lee et al., screened 50 drugs in organoid models of bladder cancer, expressing the FGF receptor, mitogen-activated protein kinase, and the mechanistic target of rapamycin inhibitors (192). More recently, Song and colleagues reported methods for generating and biobanking high-fidelity patient-derived glioblastoma organoids to test personalized therapies and model CAR T cell-based immunotherapy (179).

A less described application of organoids lies in a better understanding and prediction of treatment-related side effects, which is often observed with targeted therapy. As organoids can be generated from both healthy and tumor tissues of the same patient, they offer the possibility to screen for drugs that specifically target tumor cells while leaving normal cells unharmed thus potentially reducing toxicities in clinical trials (193).

Despite the multiple downstream therapeutic applications of tumor organoids, the lack of stromal components and of an immune-competent microenvironment may hamper the implementation of this approach in a clinical setting. Therefore significant efforts have already being made in order to incorporate aspects of the TME into the cancer organoid system and thus to decipher complex tumor immune cell crosstalks, to identify immune evasion mechanisms and to determine the effectiveness of various immunotherapeutic approaches (194).

Three main strategies have been developed to date to capture TME cell heterogeneity and heterotypic cell interactions, specifically: (*i*) reconstituted submerged cultures (195), (*ii*) holistic microfluidic 3D cultures (196), and (*iii*) air-liquid interface (ALI) cultures (197).

In reconstituted TME models, organoids containing exclusively cancer cells, derived from mechanically and enzymatically dissociated tissues, are cultured in ECM domes (*e.g.*, Matrigel™ or Cultrex^®^ Basement Membrane Extract) and submerged beneath tissue culture medium. Exact culture conditions are customized for specific tumor histologies, but often include various growth factors and/or pathway inhibitors which allow stem cells to undergo self-renewal and differentiation (*e.g.*, in intestinal organoids) (20). To model the TME, exogenous immune cells, such as those from autologous peripheral blood or tumor bulk, are isolated and subsequently co-cultured with grown organoids. Such submerged reconstituted PDOs are suitable for modeling cancer disease and for screening drug efficacy by recapitulating not only the genetic and phenotypic diversity of original tumors, but also the functional patient responses to clinical treatment (187, 198). Several reconstitution approaches were developed by supplementing PDO cultures with CAFs. Interestingly, human ductal adenocarcinoma (PDAC) organoids co-cultured with CAFs revealed that CAF-secreted Wnt drives organoid growth in Wnt-non-producing PDAC subtypes (156). Additionally, co-culture of murine pancreatic stellate cells with PDAC organoids revealed desmoplastic stroma production and heterogeneous CAF differentiation into two distinct subtypes: IL-6-expressing inflammatory CAFs activated by paracrine secreted factors from tumor cells, and high αSMA-expressing myofibroblast-like CAFs that interact with tumor cells (199). Of note, in another study reconstituted PDOs enabled the identification of IL-1 and TGFβ as tumor-secreted ligands responsible of shaping the above-mentioned CAF heterogeneity (200). Similarly, Ebbing, van der Zalm et al. co-cultured oesophageal adenocarcinoma organoids with patient-derived CAFs and found that stromal-derived IL-6 drove epithelial-to-mesenchymal transition and therapeutic resistance (201). Diverse immune cell reconstitution of submerged Matrigel™ organoids has also been performed. By co-culturing patient-matched CAFs and peripheral blood lymphocytes (PBLs) with PDAC organoids, Tsai et al. demonstrated myofibroblast-like CAF activation and tumor organoid lymphocyte infiltration (202). A noteworthy study reported a more complex setup involving a triple co-culture of mouse gastric tumor organoids, DCs and cytotoxic T lymphocytes (CTLs). In the presence of anti- PD-L1 neutralizing antibody, antigen stimulated-CTLs killed gastric tumor organoids, suggesting that the reconstitution of multiple immune cells may allow the study of tumor–immune and immune–immune cell crosstalks (203). Furthermore, reconstitution models of tumor organoids with autologous PBLs hold the potential to predict the functionality of TILs after ICB-based therapy. In a proof-of-principle study, Ramsay and colleagues co-cultured human colorectal cancer organoids with TILs and observed that exposition to anti–PD-1 antibody partially restored antitumor immunity of PD1-expressing T cells (204). Accordingly, Voest’s group generated tumor-reactive CD4^+^ and CD8^+^ T lymphocytes by co-culturing autologous PBMCs with colorectal cancer or NSCLC PDOs, in medium supplemented with IL-2, anti-CD28, and anti-PD1 (195, 205). In a clinical study with early stage colon cancer patients treated with neoadjuvant immunotherapy, Chalabi et al. used the same autologous organoid and PBMC co-culture system to potentially correlate *ex vivo* induced T cell reactivity to patient response. However, T cell reactivity could only be partly linked to clinical response, due to the absence of anti-CTLA4 in the co-culture system and lack of key TME constituents (206). Organoid-based immune assays have also been explored to provide a rationale for combination treatments of targeted MEK or BRAF inhibitors with multiple ICB agents (207).

Given their adaptability, tumor organoids have been applied for numerous other immunotherapeutic approaches. In this regard, Gonzalez-Exposito et al. used patient-derived colorectal cancer organoids to gain insight into treatment response to cibisatamab, a carcinoembryonic antigen (CEA)-targeting bispecific antibody (208) demonstrating that heterogeneity and plasticity of CEA expression conferred low sensitivity to such an agent. Moreover, tumor organoids may support studies in the field of adoptive cellular therapy (ACT), including the use of tumor TIL, NK, and CAR-T cell treatments. Intriguingly, Schnalzger et al. used available matching normal and tumor organoids to explore tumor antigen-specific cytotoxicity of CAR-NK cells (209).

Otherwise, holistic TME models preserve, as a cohesive unit, the intrinsic immune microenvironment of tumor specimens along with tumor cells. Of interest, spheroid-based organotypic cultures within collagen gels in 3D microfluidic culture devices have been adapted to culture murine- or patient-derived tumors (114). Briefly, tumor spheroids from syngeneic immunocompetent murine models and patient tumor specimens, such as melanoma and Merkel cell carcinoma, were mixed with collagen gels, injected into microfluidic devices and cultured for 1–2 weeks (196, 210, 211). Flow cytometric immune cell profiling showed that such organotypic cultures were able to retain cancer cells as well as autologous lymphoid and myeloid cell populations while recapitulating the *in vivo* therapeutic sensitivity and resistance profile to PD-1 blockade (211). *In vitro* culture systems are further being deployed to explore novel mechanisms, therapeutic combinations, and putative biomarkers relevant to ICB response and resistance. In another study, small-molecule screening identified CDK4/6 inhibitors as compounds enhancing T cell activation in PD-1-overexpressing Jurkat T cells. Combination of CDK4/6 inhibition and PD-1 blockade significantly induced tumor cell death *in vitro* in MC38 murine-derived organoids, as evidenced by tumor live/dead staining as well as by T cell-mediated tumor growth inhibition *in vivo* in syngeneic MC38 and CT26 mouse models (210).

More recently, ALI cultures offer a valuable and more sophisticated alternative to co-culture the original tumor epithelium *en bloc* with its native stromal and immune cells without any reconstitution (212). In this method tumor organoids from minced primary tissue fragments containing both tumor cells and immune components are embedded in a collagen gel within an inner transwell dish. Culture medium in an outer dish diffuses *via* the permeable transwell into the inner dish and the top of collagen layer is exposed to air *via* an ALI, allowing cells access to a sufficient oxygen supply (197). Initially, ALI organoid method was developed to culture different normal tissues, including small intestine, colon, stomach, and pancreas, which were shown to comprise both epithelial and mesenchymal components. Subsequently, this technology was extended to the establishment of PDOs from human biopsies, such as melanoma, renal cell carcinoma, and non-small cell lung cancer, as well as from murine tumors in syngeneic immunocompetent mice (197). ALI PDOs preserve not only the genetic alterations of the original tumor, but also the complex cellular composition and architecture of the TME. Indeed, both tumor parenchyma and stroma are retained, including fibroblasts and a variety of endogenous infiltrating immune cell populations, such as TAMs, T cells [T helper (Th), cytotoxic (Tc), regulatory (Treg), and exhausted (Tex)], NK cells, and B cells (197).

Strikingly, the ALI PDOs could preserve the T cell receptor (TCR) heterogeneity found in the original tumor and model immune checkpoint-dependent mechanisms of immune suppression (197). Indeed, ALI organoids grown from mouse tumors inoculated into syngeneic immunocompetent mice (*i.e.*, B16-SIY, MC38, and A20) and from diverse human cancer biopsies, (such as NSCLC, melanoma, and renal cell carcinoma) exhibited antigen-specific clonal CD8^+^ T cell expansion, activation and subsequent tumor killing in response to anti-PD-1/PD-L1 antibodies (213).

As extensively discussed, tumor organoids have undoubtedly emerged as physiological relevant *in vitro* models to study cancer biology. However, to realize their full potential, key challenges need to be addressed. First, the use of non-standardized and ill-defined culture protocols across cancer organoid studies (*i.e.*, cancer tissue source, medium formulations, animal-derived 3D matrices) introduces a huge technical variability that leads to a misrepresentation of cancer’s intrinsic biological heterogeneity which may potentially affect drug development and biomarker discovery (214). Second, for several cancer subtypes the efficiency of organoid derivation is extremely low and to date only few studies were able to adapt the organoid approach for non-epithelial cancers (11, 127). Third, established organoid cultures often include only cancer cells and do not support long-term co-culture of other TME cell types (212). In the future, the development of next-generation tumor organoids will require a meticulous patient-specific understanding of the *in vivo* tumor niche in order to identify the necessary medium components to maintain non-neoplastic cells in culture and favor heterotypic interactions. Last, but not the least, organoids are significantly high time- and resource-consuming and in order to become highly relevant model for translational applications they require optimization of high-throughput and high-content functional readout analyses, as already described for spheroids.

### 3.3 Tumor-on-a-Chip models

Organ-on-a-Chip (OoC)-technology is a rapidly evolving, highly innovative, and promising tool that allows *in vitro* microscale biomimetics of human organs. By flanking and integrating cell biology with microengineering and microfluidics, OoCs model physiological and pathological tissue microenvironments thus breaking conventional *in vitro* and *in vivo* impasses (215). Specifically, OoCs are multichannel microfluidic cell-culture devices hosting multiple cell types organized in a 3D tissue, and even organ, structure in order to model with high fidelity, and to control with high precision, key structural and functional units including, but not limited to, vasculature-like perfusion, heterotypic cellular interactions, flows of chemical gradients and mechanical forces (216–219). These features make OoCs accurate human-relevant models critical to address questions that conventional cell culture and animal models do not (220, 221). Indeed, conventional *in vitro* models are not complex enough to recapitulate tissue/organ pathophysiology, and animal models do not faithfully mimic human disease and natural and therapy-induced response (218). Since their first introduction in basic research in the early 2000s (222–224), OoCs rapidly became a valuable asset to model and dissect a wide range of pathologies across all human organs (225–231) as well as to screen and test various therapeutics (232, 233). For the sake of completeness, as the OoC field is constantly evolving, new devices with improved functionality, integration, and automation are emerging that recapitulate multi-organ-, body-, and even patient-on-chip complexities at once, as exhaustively covered in (234–237). Therefore, the key advantage of OoCs is the unique possibility, they offer, to recreate a patient-tailored disease model taking into consideration the genetic make-up, sex and gender features that affect drug response (238). Of note, OoCs enable high-throughput, high-resolution, live imaging, which allows to track cell trajectories and quantify heterotypic cell interaction times at once. In addition, end point assays could be performed to interrogate recovered cell states at transcriptomic, proteomic and biochemical/metabolic levels and to analyze cell secretome on perfused cell culture media (239). All these aspects render OoC platforms perfect tools for cancer research, which led to the rise of the ToC concept (202, 240–242) (**Table 1**). Today we dispose of state-of-the-art ToC platforms that allow, in a less than 1-inch chip, to precisely recapitulate and timely control critical hallmarks of the TME and to integrate all tissue components while envisioning in real-time cell-to-cell interactions and co-evolutions (243, 244). In particular, immunocompetent ToC (iToCs) models are emerging as precious tools to analyze and manipulate crucial aspects affecting both cancer onset and progression as well as response to therapy (245, 246). Indeed, 2D cell culture models do not recapitulate the TME and *in vivo* animal models do not effectively resemble its immune contexture and the response to immunogenic and immune-based therapies (247, 248). By *in vitro* mimicking human immunity, advanced iToCs address these unmet challenges and help understand natural and therapy-induced evolutive pressures as well as predict the clinical efficacy and safety outcomes of tested drugs (245). In addition, by integrating TME biomimicry with vasculature-like perfusion, iToCs allow to tightly control and manipulate oxygen and nutrient supply, the release of growth factors and cytokines and the interaction with ECM components (249, 250) and to recapitulate and systematically depict the communication between cells in disease progression and metastatic dissemination in an unprecedented detail (251, 252). First generation iToCs were designed to represent study-tailored TME where cell types and positions reflect experimental needs and specific research questions. In their simplest form, iToCs have been used to study 2D cell migration and immune cell chemotaxis in response to chemokine and immune alarmin gradients (246, 253–257). Immune cell trajectories and interaction with cancer cells were real-time monitored and quantified by time-lapse microscopy and automated tracking analysis (246, 254–257). In a bit more complex system, we and others analyzed competitive immune chemoattractant forces of cancer cells with diverse immunogenicity by culturing in opposite, microchannel connected, chambers three different cell types (246, 254, 257, 258). Similar devices have been used to study cancer cell interactions with stromal and immune cells when cultured in separate chambers (259). In a seminal study, Yu and collaborators described a reconfigurable iToC allowing the spatiotemporal control of paracrine signaling between pancreatic cancer cells and TAMs (260). According to a fit-for-purpose approach, the authors assembled a ‘stackable’ multi-culture system, in which each cell component was cultured in a distinct layer, and then stacked, unstacked, and dynamically reconfigured over the course of the study in order to control the spatial and temporal interaction of these subsets. By manoeuvering the system, the authors recapitulated the *in vivo* observation that paracrine signaling from more aggressive prostate cancer cell variants tips the balance of TAM polarization toward an anti-inflammatory, M2-like phenotype which, in turn, promotes the formation of new blood vessels by signaling with endothelial cells (260). Similar findings were described by Guo et al. and Kim et al. in a NSCLC and triple-negative breast cancer (TNBC) model, respectively (260–262). As ECM meshwork is known to deeply condition cancer immunosurveillance and therapeutic response (263–265), more complex iToC systems including ECM scaffolds were developed that allowed researchers to create a 3D TME where testing with high fidelity the therapeutic effects of immune-based drugs (266–269). More recently, *ex vivo* cultures of human tumor tissues were introduced in iToCs as a representative platform to profile ICB-based therapies (211). Notably, within this setting, elaborated iToCs have been designed that are characterized by a tightly planned spatial compartmentalization allowing immune cell migration from lymphnodes to blood circulation up to the TME, with the intent to faithfully reproduce the human cancer-immunity cycle (245, 270, 271). In particular, Shim et al. established a first model of multi-compartment iToC that recapitulates lymphnode-tumor bulk crosstalks through a continuous perfusion of culture medium (272). In the effort to integrate measurements of additional chemical and physical cues (*e.g.*, oxygen levels, cytokine and chemokine flows, ECM stiffness and remodeling, lymphatic shear stress and blood perfusion, among the others) working in the TME, *in vitro* iToCs have been combined with multiscale *in silico* modeling (273, 274). This systematic analysis offers a panoply of combined factors and dynamics molding overall tumor behavior in terms of progression and therapeutic response (273, 274). Specifically, oxygen levels in ToC models have been tuned either by the introduction of physical barriers (266), or by placing the device into an hypoxic, adjustable culture chamber (266), or by naturally generating hypoxic cores within cancer spheroids (275). Cytokine-, chemokine- and alarmin-based flow gradients can be either pre-established in dedicated device sinks (276), or naturally triggered by the addition of soluble factors (277), or cancer cell death inducers (246, 257). As ECM composition and stiffness were shown to play a role in immune cell infiltration and therapeutic response (278), cancer matrices with diverse composition, porosity and density have been used and the ability to either impede immune cell migration, or promote immune-cancer cell crosstalk have been tested (266, 279). Lymphatic and blood perfusion are crucial players within the TME as they affect immune cell homing and cancer cell diffusion to distant sites (280–282). However, conventional *in vitro* cancer models almost always lack vascular perfusion. In this sense, iToCs represent a significant step forward, as they inherently include vessel-like microchannels that researchers took advantage of for studying neo-angiogenesis (283), cancer cell spreading through intravasation (284) and extravasation (285), and off-target effects of anticancer treatments (286). Moving forward in complexity and fidelity, through careful study design and co-culture selection, researchers have integrated cancer spheroids and organoids in iToC models (219, 287, 288). Chemical and biological drug testing are the most promising applications of cancer organoids-on-a-Chip as this merger greatly improves the fidelity of TME *in vitro* reconstruction. Hence, on the one hand cancer organoids, as described above, are miniaturized tumors that follow intrinsic developmental programs, developing from self-organizing stem cells, and resembling their *in vivo* counterparts better than any other *in vitro* modeling, on the other hand microfluidic platforms are man-made constructs in which heterotypic cell components and their microenvironment are precisely controlled (288). Moving forwards, the design of multi-organoids in iToCs could open the possibility to test drugs on patients routinely excluded from clinical trials, such as children and pregnant women. However, despite the impressive advances in iToC field and the enormous potential these models offer, issues remain that need to be addressed to reach the ambitious goal to broadly apply iToCs in biomedical research (289, 290). Indeed, it is still not possible to assess some systemic drug toxicities and side effects currently studied using animal models (*e.g.*, vomiting, diarrhea and alopecia). Integration of *in silico* modeling and artificial intelligence-based data analysis could maybe help in this sense and circumvent this limitation to some degree. Moreover, iToCs need to be improved in terms of throughput, adaptability and manufacturing. Indeed, (*i*) the high *in vivo* relevance of these models comes at the price of low throughput, as only a few replicates can be performed at once; (*ii*) the culture microenvironment needs to be adapted to the according patient-derived tumor, in a fit-for-purpose approach; and (*iii*) the high cost and the availability of equipment and materials to realize iToCs are a challenge to scale-up the manufacture (289–291).

**Table 1:** Summary of Tumor-on-a Chip platforms.

Tumor-on-a-Chip models	Cell types	Applications	Drugs	References (PMID)
Lung Cancer	A549, H1975, H560, LCA-1, NCI-H1650, H2052 spheroids, BE063-T, BE069-T spheroids	drug response and resistance, evaluation of photodynamic therapy, tumor-stroma crosstalks, tumor-bacteria crosstalks, tumor migration and metastasis	Gefitinib, Afatinib, Osimertinib, Erlotinib, Cisplatin	36005014, 29029734, 29020635, 26088102, 29686328, 27606718
Breast Cancer	ductal carcinoma in situ cells, MCF10A, HMT-3522, BC tumor organoids, MDA-MB-231, SUM-159, SK-BR-3 spheroids	tumor invasiveness and metastasis, angiogenesis, cell cytotoxicity, drug sensitivity, metabolic adaptation	Doxorubicin, Tirapazamine, Paclitaxel, Taxol	27549930,30482722, 30723584, 30393802, 27678304, 33094918, 36278146
Prostate Cancer	DU145, LNCaP, C4–2, PC3, BCaP	TME mimicking, immunological studies, drug testing	Docetaxel, Paclitaxel	30810874, 28371753, 33034643, 31427781
Colorectal Cancer	CRC-268, Caco-2, HCT116, SW620, SW480, HT-29, MC38 spheroids, CT26 spheroids, colon organoids	angiogenesis, tumor-stroma crosstalks, immunological studies, drug sensitivity, nanomedicine, pharmacokinetic, pharmacodynamic, tumor metastasis	Bevacizumab, FOLFOX, Oxaliplatin, Pazopanib, Vincristine, CMCht/PAMAM dendrimer nanoparticles loaded with gemcitabine, Doxorubicin, Pembrolizumab, Ipilimumab, 5-fluorouracil	30393802, 27549930, 31131324, 28544639, 27796335, 27391808, 28439087, 20126684, 29101162, 34113836
Pancreatic Cancer	PAC, PANC-1, PDAC162, PDAC175, PD7591, MH6883, PD883, MH6556, S2-028, KPC2, eKIC, mKIC, PDOs	immunological studies, TME mimicking, EMT investigation, drug resistance, tumor-stroma crosstalks, tumor invasiveness	Gemcitabine, All-trans retinoic acid, Clodrosome, Paclitaxel	32930334, 31489365, 31546820, 31997571, 35450328, 29329547
Melanoma	MNT-1, WM-115, LOX-IMVI, A-375, SK-Mel-28 spheroids	angiogenesis, tumor-stroma crosstalks, tumor migration, drug resistance, immunological studies	Vemurafenib	33533390, 26542093, 36671624
Ovarian Cancer	SK-OV-3, OV90, OVCAR-3,A2780	angiogenesis, tumor-stroma crosstalks, TME mimicking, immunological studies, tumor invasiveness, tumor-platelet crosstalks, tumor metastasis	Cisplatin, Revacept	28544639, 34290095, 32851999, 33524968, 35995621
Brain Cancer	U-251 MG, primary GBM cell culture, U87 spheroids, GS5, SKNBE, PC9-BrM3	TME mimicking, drug screening, tumor cell heterogeneity, tumor metastasis	Temozolomide, Tirapazime, Cisplatin, Irinotecan, Isotretinoid	31148598, 27151082, 27796335, 31016107, 29158813, 31034948

TME, tumor microenvironment; EMT, epithelial-to-mesenchymal transition; PDO, patient-derived organoid; GBM, glioblastoma.

In sum, while ToC models are still unable to fully replace animal studies, the ever growing flow of innovation in the design and development of microfluidic iToC technologies will continue to provide ripe rewards for the cancer research and will help to solve unmet challenges in both basic biology and clinical patient management, particularly in the field of immune-oncology and cancer immunotherapy, as comprehensively reviewed in (245).

## Concluding remarks

The improvement and integration of cancer spheroids, organoids and iToCs into cancer research, drug development pipelines and patient care hold great potential as these models offer biologic fidelity along with experimental control as never before. Hence, 3D cancer models help recreate, in a stepwise manner, the complexity of the TME by making possible to decipher, monitor and timely maneuver the roles of individual cell players and of their reciprocal interactions on tumor progression and (immune)therapy response. Joining forces, know-hows and skills from micro-engineers, cancer immunologists, pharmaceutical researchers and bioinformaticians is anticipated to achieve the ambitious goal of overcoming the near-term challenges of these platforms in order to expand their implementation in disease modeling and drug discovery. Our vision is that, the selection of the right 3D cancer model for each experimental purpose, and the proper reconstitution and handling of the immune system, allow the development of integrated high fidelity TME representations and help to explore fundamental biology, and to tackle key issues of drug testing with critical impact on clinical management. This ultimately will help refine, reduce and replace animal studies, while helping human patients.

## Author contributions

MM conceived the review. MM, AS and RD wrote the manuscript with constructive input from all authors. NM and EM prepared display items under the supervision of MM. All authors contributed to the article and approved the submitted version.
